# Synergy of Cellulase Systems between *Acetivibrio thermocellus* and *Thermoclostridium stercorarium* in Consolidated-Bioprocessing for Cellulosic Ethanol

**DOI:** 10.3390/microorganisms10030502

**Published:** 2022-02-24

**Authors:** Na Wang, Zhihua Yan, Na Liu, Xiaorong Zhang, Chenggang Xu

**Affiliations:** Key Laboratory of Chemical Biology and Molecular Engineering of Ministry of Education, Institute of Biotechnology, Shanxi University, Taiyuan 030006, China; nawangsxu@163.com (N.W.); yzhsxtyyx@163.com (Z.Y.); lnzbstaiyuan@163.com (N.L.); zxrtaiyuan@163.com (X.Z.)

**Keywords:** enzymatic activity, synergy, co-fermentation, consolidated bioprocessing, *Acetivibrio thermocellus*, *Thermoclostridium stercorarium*

## Abstract

Anaerobes harbor some of the most efficient biological machinery for cellulose degradation, especially thermophilic bacteria, such as *Acetivibrio thermocellus* and *Thermoclostridium stercorarium*, which play a fundamental role in transferring lignocellulose into ethanol through consolidated bioprocessing (CBP). In this study, we compared activities of two cellulase systems under varying kinds of hemicellulose and cellulose. *A. thermocellus* was identified to contribute specifically to cellulose hydrolysis, whereas *T. stercorarium* contributes to hemicellulose hydrolysis. The two systems were assayed in various combinations to assess their synergistic effects using cellulose and corn stover as the substrates. Their maximum synergy degrees on cellulose and corn stover were, respectively, 1.26 and 1.87 at the ratio of 3:2. Furthermore, co-culture of these anaerobes on the mixture of cellulose and xylan increased ethanol concentration from 21.0 to 40.4 mM with a high cellulose/xylan-to-ethanol conversion rate of up to 20.7%, while the conversion rates of *T. stercorarium* and *A. thermocellus* monocultures were 19.3% and 15.2%. The reason is that *A. thermocellus* had the ability to rapidly degrade cellulose while *T. stercorarium* co-utilized both pentose and hexose, the metabolites of cellulose degradation, to produce ethanol. The synergistic effect of cellulase systems and metabolic pathways in *A. thermocellus* and *T. stercorarium* provides a novel strategy for the design, selection, and optimization of ethanol production from cellulosic biomass through CBP.

## 1. Introduction

Lignocellulosic biomass is the most abundant renewable resource on earth, yet its structural complexity has hampered its exploitation for cellulosic ethanol and biochemicals. Efficient conversion of lignocellulosic biomass to liquid transport fuels such as ethanol is one of the most promising and important methods among alternatives to fossil fuels because of its potential sustainability, security, and rural economic benefits [1]. During the past few years, commercial cellulosic ethanol has obtained global attention with developments happening quickly in Brazil, USA, China, Italy, India, and Spain [2,3], but the process technology used in these industrial installations is still under development. In addition, the economic production of cellulosic ethanol at large scale is also still a challenge, such as generation of inhibitors by pre-treatment, expensive enzyme cost and co-utilization of xylose [4,5,6]. CBP, in a single vessel or reactor with low process complexity, which simultaneously combines lignocellulosic biomass hydrolysis and fermentation of full-spectrum of the liberated sugars, is a promising strategy in energy conversion by reducing biological processing costs and an economical approach to the production of cellulosic ethanol [7,8]. However, no natural “CBP organism” that combines all these features has been identified so far [8,9]. The classical organism used for ethanol production, *Saccharomyces cerevisiae*, is not suitable for cellulosic ethanol production via CBP because of its inability to survive at the optimal temperatures of exogenous cellulases, pentose fermentation, and cellulose degradation [10].

Cellulolytic and thermophilic microorganisms has been proposed as a suitable CBP host for the production of second-generation biofuels [11]. Among the organisms with CBP capability, *Acetivibrio thermocellus* (previously *Clostridium thermocellum*) has attracted growing interest due to its cellulolytic and ethanologenic properties under thermophilic conditions in recent years [12,13]. In addition, it has a developed genetic transformation system that allows for the construction of mutant strains [14,15]. To solubilize lignocellulose, *A. thermocellus* secretes an extracellular multi-enzyme complex termed as the cellulosome that is central to *A. thermocellus*’s ability to reduce recalcitrance of lignocellulosic biomass, which is an excellent CBP candidate due to its remarkable cellulose solubilization ability [16,17,18]. Another thermophilic and cellulolytic anaerobe, *Thermoclostridium stercorarium* (previously *Clostridium stercorarium*), which is phylogenetically closely related to *A. thermocellus* (both of them are in Family 4, Genus 2 of Clostridia [19]), can also efficiently degrade polysaccharides in plant biomass and produce acetate, ethanol, CO_2_, and H_2_, thus resulting in lower ethanol yields [20,21]. However, the cellulase system of *T. stercorarium* is of a “free” form, not the typical “complex” form of anaerobes such as *A. thermocellus*. *T. stercorarium* has only two main cellulases: Avicelase I (CelZ) and Avicelase II (CelY), which are respectively orthologous to Cel9R and Cel48S from *A. thermocellus*, which are the most important during hydrolysis of crystalline cellulose hydrolysis as representatives of GH48 and GH9 family cellulosomal components [22,23]. Meanwhile, *T. stercorarium* has a great number of hemicellulases, suggesting that it is especially suitable for fermentation of hemicellulose to organic solvents [19,21,24].

The major disadvantage of *A. thermocellus* for fermentation of lignocellulosic biomass is lack of the ability of utilizing pentoses, while *T. stercorarium* grows well on xylan, soluble cellodextrins, glucose, xylose, arabinose, fructose, galactose, mannose, and ribose. Therefore, we are intrigued by the possibility of establishing a co-culture system of *A. thermocellum* with *T. stercorarium* can improve substrate utilization and ethanol yield due to synergistic effects. In this study, we firstly compared the composition and substrate specificities of two individual cellulase degradation systems, *A. thermocellus* LQR1 and *T. stercorarium* ATCC 35414. Furthermore, the synergistic effect of these two systems on cellulose degradation was investigated. When the two thermophilic and cellulolytic strains were co-cultured using the mixture of cellulose and xylan as the substrate, faster cellulosic hydrolysis, higher ethanol yield, and higher cellulose-to-ethanol conversion rate than the monoculture were observed. Thus, fermentation using co-culture of *A. thermocellus* and *T. stercorarium* is an effective way to be employed in CBP for achieving improved ethanol production from cellulose.

## 2. Materials and Methods

### 2.1. Strains, Media, and Culturing Conditions

*Acetivibrio thermocellus* (At) LQR1 was kindly provided by Prof. Jizhong Zhou, University of Okalahoma, USA. *Thermoclostridium stercorarium* (Ts) ATCC 35414 was purchased from the American Type Culture Collection. Both Ts and At were cultured anaerobically at 60 °C in mineral medium supplemented with 0.2% cellobiose for cellulases extraction and in GS-2 medium [25] supplemented with mixture of 0.6% Avicel (PH-101, Sigma, Burlington, MA, USA, <50 μm) and 0.4% xylan for ethanol fermentation.

### 2.2. Cellulase Systems Isolation

**Figure A1:**
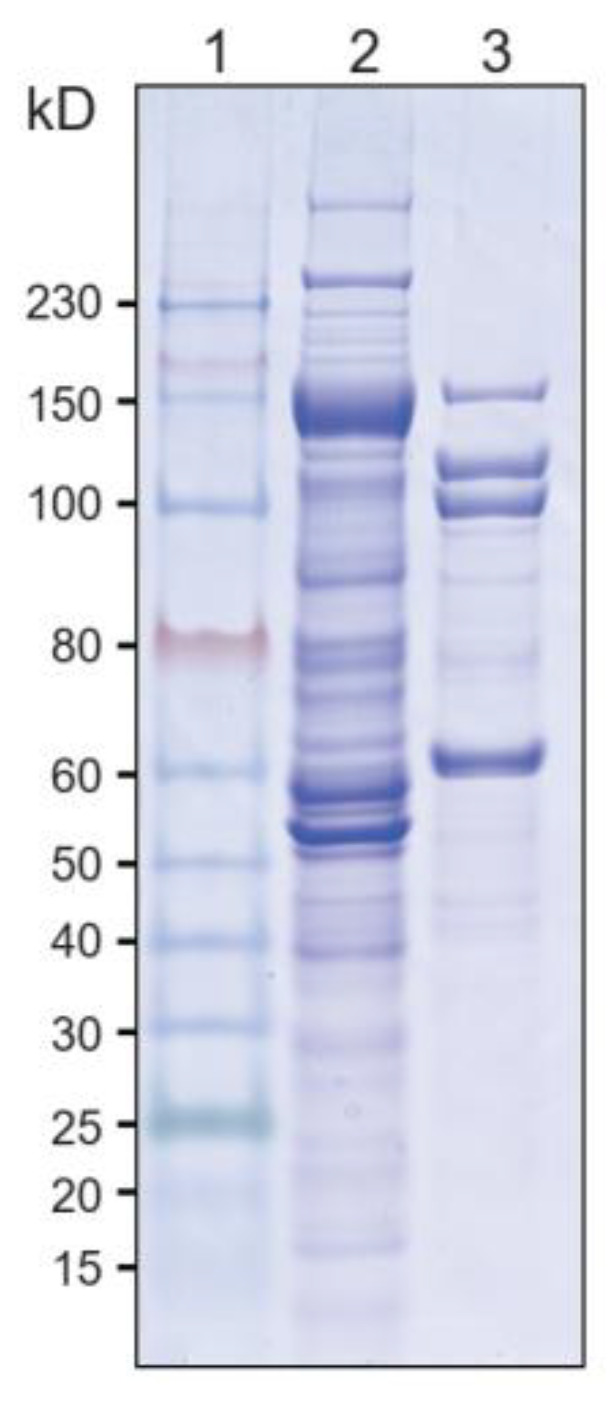
SDS-PAGE of the cellulase systems from *A. thermocellus* and *T. stercorarium*. Lane 1, protein marker; Lane 2 and 3, the cellulase systems from *A. thermocellus* and *T. stercorarium*, respectively.

At and Ts were grown in 1 L of 0.2% cellobiose-supplemented mineral medium for 48 h, respectively. Then the cellulase systems in At and Ts were isolated from the cell-free fermentation broth through a stirred ultrafiltration cell equipped with a polyethersulfone ultrafiltration membrane (10-kD cutoff, Millipore, Billerica, MA, USA) and dialyzed with MES buffer (50 mM MES, 5 mM CaCl2, pH6.0). The samples were detected by NuPAGE^®^ Bis-Tris gel with MOPS SDS Running Buffer (Invitrogen, Waltham, MA, USA) (Figure A1). The total protein concentration of the isolated enzymes samples was determined by a Bradford assay. The protein standard was bovine serum albumin (Sigma, Burlington, MA, USA). The enzymes were stored at −20 °C prior to further analysis.

### 2.3. Enzyme Activity Measurement

Glycoside hydrolase activities on polysaccharides were assayed by incubating 0.1 mg/mL enzymes in an assay mixture containing 1% (*w*/*v*) of Avicel PH101, carboxymethyl cellulose (CMC), phosphoric acid-swollen cellulose, filter paper (waterman No. 1), oat spelt xylan, citrus peel pectin, or milled corn stover in MES buffer at 60 °C for 24 h. The released sugar concentration was estimated by the dinitrosalicylic acid (DNS) method using glucose as the standard [26] at time points of 0, 1, 2, 4, 8, and 24 h. Phosphoric acid-swollen cellulose was prepared as described previously [27]. Cellobiosidase activity was measured using cellobiose as the substrate in above reaction systems. The concentration of generated glucose was estimated by the D-glucose kit (Megazyme, Wicklow, Ireland) following the manufacturer’s instructions. All experiments were performed in triplicate.

The unit (U) of enzyme activity is defined as the amount of cellulases required to release 1 μmol “reducing sugar” in 1 h from polysaccharides at 60 °C [28].

### 2.4. Synergistic Interaction Assay

Examination of the synergistic effect between At and Ts was conducted using 1% Avicel or corn stover as the substrate in MES buffer and with an incubation time of 12 h at 60 °C. Both cellulase systems were diluted to equal protein concentrations and combined at different ratios in the assay mixture to maintain a constant sum (0.1 mg/mL) of individual protein concentration. Cellulase systems of At and Ts were tested individually as controls. The released sugar concentration was estimated by the DNS method. The degree of synergy is the ratio of sugar produced when both enzyme systems are present to the sum of the sugar produced when they are present individually [29,30].

Sugar profiles produced by cellulase systems were analyzed using ion chromatograph ICS-3000 (Dionex, Sunnyvale, CA, USA) equipped with a 4 mm × 250 mm CarbopacTM PA 20 column (Dionex, Sunnyvale, CA, USA).

### 2.5. Fermentation and Analytical Methods

The seed culture of At and Ts was prepared in a GS-2 medium supplemented with 0.2% cellobiose in the later log phase, and 1:100 inoculated into 200 mL N2-flushed GS-2 medium containing 0.6% Avicel (6 g/L) and 0.4% Xylan (4 g/L), respectively. Meanwhile, co-fermentation of At and Ts was performed under the same condition with an equal inoculation amount of At and Ts, and the final inoculation amount was same as the individual fermentation. They were grown anaerobically at 60 °C for seven days and sampled at different time points to measure concentrations of residual cellulose, xylan, and reduced sugar. At the end of each experiment (Day 7), the organic acid and ethanol yield in the medium were measured. The conversion rate of substrate to ethanol was calculated by the percentage of ratio of ethanol yield to polysaccharide (Cellulose and/or xylan) consumption (*w*/*w*). All experiments were performed in triplicate.

The colorimetric DNS method was used to measure the concentrations of reducing sugar [31]. The cellulose and xylan amounts were determined by the sulfuric acid hydrolysis method [32,33]. The ion chromatography system ICS-3000 (Dionex, Sunnyvale, CA, USA) equipped with a 4 mm × 250 mm IonPac^®^ AG11-HC column (Dionex, Sunnyvale, CA, USA) was used to measure concentrations of formic, acetic, and lactic acid by comparing with their standard solutions (Merck, Darmstadt, Germany). Ethanol concentrations were quantified using the enzymatic K-Ethanol kit (Megazyme, Wicklow, Ireland) according to the manufacturer’s protocol.

## 3. Results

### 3.1. Comparison of Components of Cellulase Systems between A. thermocellus and T. stercorarium

We compared the components of glycoside hydrolase (GH) family enzymes of both *A. thermocellus* (At) and *T. stercorarium* (Ts) systems according to the CAZy database (www.cazy.org (accessed on 18 February 2022)) [34,35]. The results indicated that At (72 CAZymes in 29 GH families) and Ts (68 CAZymes in 37 GH families) possess almost equal numbers of genes encoding GH family enzymes, but they are significantly different in terms of enzyme type and quantity in each GH family (Table 1).

Firstly, 50 genes harboring dockerin domain, unmistakable conserved sequences for cellulosomal components, are found in At, but none in Ts, suggesting that the cellulase systems between At and Ts are very different regarding organization structure. Secondly, GH9 and GH48 are the most important cellulases for crystalline cellulose hydrolysis [22]. Combining the GH48 cellobiohydrolase with GH9 processive endoglucanase can achieve the highest degree of synergy [34,35]. At has sixteen GH9 and two GH48 cellulases, of which fifteen GH9 and one GH48 cellulases belong to cellulosomal components, while Ts has only one GH9 and one GH48 enzyme, named Avicelase I and II, respectively (Table 1). Furthermore, At contains ten endoglucanases from GH5, which is another key family of cellulose degradation, but Ts has none. These results indicated that At harbors a much higher capacity for cellulose degradation than Ts. Thirdly, there are many more hemicellulases in Ts than in At, such as α-galactosidases (GH4 and GH36), β-galactosidases (GH2 and GH35), α-glycosidases (GH13), α-glucuronidase (GH67), and α-L-rhamnosidase (GH78) (Table 1). It suggested that Ts is especially suited for the degradation of hemicellulose. Generally, the information of genome annotation implies that At and Ts have separately evolved regarding to cellulosomes and free enzymes targeting crystalline cellulose and hemicellulose, although they have a closely related evolution relationship.

### 3.2. Substrate Specificities of Cellulase Systems from A. thermocellus and T. stercorarium

**Figure 1:**
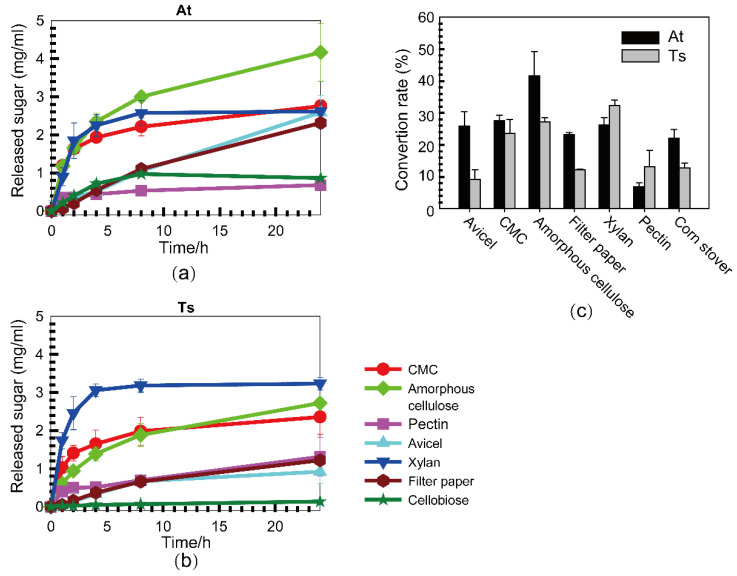
Comparison of substrate specificities of the cellulase systems from *A. thermocellus* and *T. stercorarium*. The released sugars from various substrates by cellulase systems from At (**a**) and Ts (**b**) were, respectively, measured during 24 h of incubation. The conversion rate of substrates was calculated at the end of experiments (**c**). Error bars indicate the standard deviation of the mean from experiments performed in triplicate.

We compared cellulase activities of the cellulase systems from the two cellobiose-grown strains, At and Ts, on different polymeric substrates (Figure 1a,b). The results indicated that both cellulase systems had ten times higher activity when using CMC and amorphous cellulose as substrates than when using microcrystalline cellulose, filter paper, suggesting that the speed of degradation accelerated with the decreasing polymerization of cellulose (Table 2). Additionally, the active time of cellulase was longer than that of hemicellulase, except when using CMC as the substrate. The yield of released sugar on Avicel and filter paper kept growing during 24 h of incubation, while the yield on xylan and pectin was almost up to the maximum at 8 h (Figure 1). Thus, it is suggested that hemicellulases are more exposed to the consequences of product inhibition than cellulases. Furthermore, both of them have low activity on cellobiose, i.e., activity of β-glucosidase, which catalyzes hydrolysis of cellodextrins and cellobiose releasing glucose (Table 2). It is consistent with the physiological characteristics of At and Ts, preferring to utilize cellodextrins and cellobiose rather than glucose. Meanwhile, the final conversion of various substrates after 24-h incubation was calculated (Figure 1c). It was indicated that the conversion of cellulosic substrates including corn stover by At was higher than that of Ts, while the conversion of hemicellulosic substrates, such as xylan and pectin, by Ts was higher than that of At.

On the other hand, the enzymatic activity of At and Ts cellulase systems was determined. It indicated that the cellulase activity (on Avicel, CMC, amorphous cellulose, filter paper, and cellobiose) of At is higher than that of Ts. For example, the activity of At on Avicel is 0.05 U/mg, which is 2.38 times higher than that of Ts. By contrast, the hemicellulase activities of Ts on xylan and pectin, being 0.8 and 0.185 U/mg, respectively, is higher than those of At, which are 0.414 and 0.162 U/mg, respectively (Table 2). It was suggested that the cellulase system of At is more complicated and has higher cellulose degrading ability, while the cellulase system of Ts is simpler and has higher hemicellulose degrading ability [19,36].

### 3.3. The Synergistic Effect of Cellulase Systems between A. thermocellus and T. stercorarium

Because the cellulase systems of At and Ts are different from enzymatic structure and activity, we proposed that cellulase systems between At and Ts have the synergistic effect. To analyze the synergism of the cellulase systems between At and Ts, the activity of mixed enzymes was investigated by combination of them at different ratios. The degree of synergy is estimated by the ratio of sugar produced when both enzyme systems are present for 12 h to the sum of the sugar produced when they are present individually with the same concentrations as in the mixed system [29].

**Figure 2:**
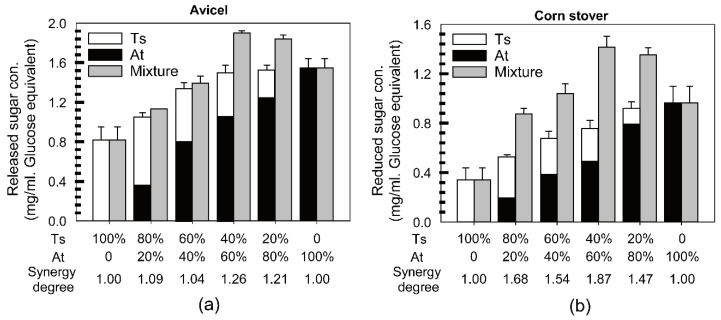
Synergistic interaction between cellulase systems from *A. thermocellus* and *T. stercorarium*. Both enzymes were separately diluted to equal protein concentration and mixed at different ratios to maintain a constant sum of protein concentration (0.1 mg/mL). Reducing sugar produced (glucose equivalents) from Avicel (**a**) and corn stover (**b**) by binary mixtures were measured after 24 h of incubation at 60 °C, and the two individual enzymes with the same amount were the controls for each ratio. The degree of synergy was defined as the ratio of sugar produced by the mixtures to the sum of the sugar produced by two individual enzymes. All experiments were performed in triplicate and are shown with standard deviations.

**Figure A2:**
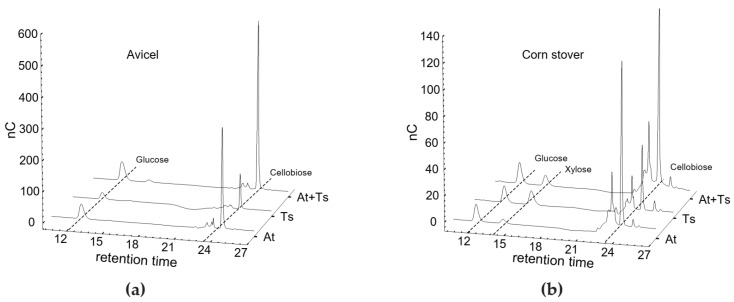
Product profiles of the cellulase systems from *A. thermocellus* and *T. stercorarium* against Avicel (**a**) and corn stover (**b**) measured by HPLC. Sugars represented by each peak were shown.

First, Avicel was used as the substrate (Figure 2a). The result showed that cellulase systems could work synergetically to degrade Avicel with the maximum synergy degree of 1.26 when the ratio of At to Ts was 3:2 after 12 h of incubation time. Then, the sugar profiles of the samples with highest synergy degree were investigated and compared with individual cellulase systems using high-performance liquid chromatography (HPLC). The hydrolysis products of Avicel were mainly glucose and cellobiose in these samples (Figure A2a), and the At and Ts combined mixture produced more sugars than At or Ts alone, especially cellobiose whose concentration in samples with mixed enzymes is, respectively, 1.32 and 3.56 times higher than that of At and Ts alone (Table 3). The synergistic effect of these two systems could be caused by their different cellulase styles, which are ‘complex’ in At and ‘free’ in Ts.

Second, corn stover was used as the substrate (Figure 2b). The maximum synergy degree reached 1.87 when the ratio of At to Ts was 3:2 as well. The hydrolysis products of corn stover are mainly glucose, cellobiose, and xylose (Figure A2b). Glucose and cellobiose were produced in larger quantities by mixture of At and Ts than At and Ts alone, which is consistent with the results of Avicel. For example, the concentration of cellobiose released by mixed enzymes is 3.41 times higher than that of Ts alone. On the other hand, the concentration of xylose released by the mixture of At and Ts is 0.71 mM, which is twice higher than that of At alone (Table 3). The results indicate that the cellulase systems from At and Ts for corn stover degradation are complementary to each other in terms of structures (between cellulosome and noncellulosome) and functions (between cellulase and hemicellulase).

### 3.4. Co-Fermentation of A. thermocellus and T. stercorarium with a Mixture of Cellulose and Xylan

**Figure 3:**
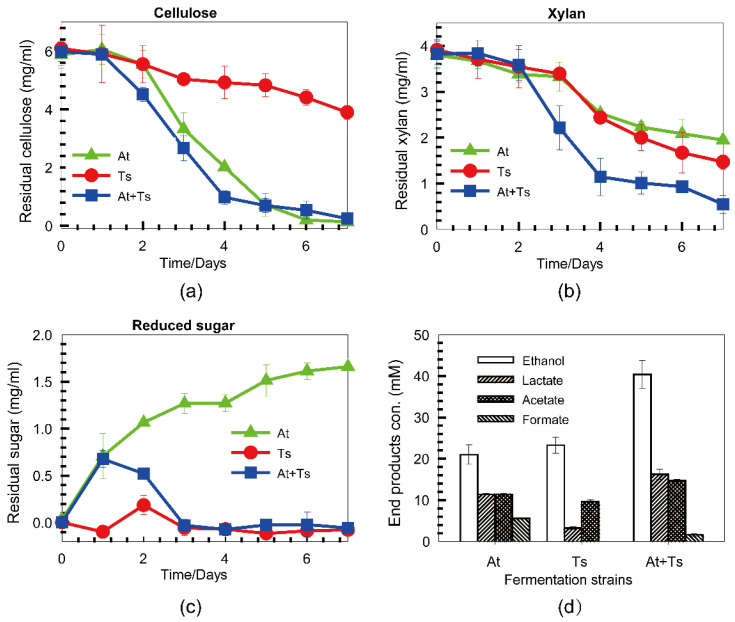
Co-fermentation of *A. thermocellus* and *T. stercorarium* with a mixture of cellulose and xylan. Co-cultures of *A. thermocellus* and *T. stercorarium* were grown in GS-2 medium supplemented with a carbon source of mixture Avicel and xylan with the monoculture of *A. thermocellus* and *T. stercorarium* as control. Cellulose (**a**), xylan (**b**) and residual sugar (**c**) were measured every day and the organic acid and ethanol yields in culture broth were measured at the end of fermentation (**d**).

To further probe the implication of the synergistic effect between two cellulase systems on cellulosic ethanol production, the performance of co-culture of At and Ts were studied. At and Ts were co-fermented with a mixture of cellulose (6 g/L) and xylan (4 g/L) as the substrates, while At and Ts were fermented individually as controls. The concentrations of cellulose and xylan against time were illustrated as Figure 3a,b, respectively. It was showed that the hydrolysis rate of cellulose and xylan is significantly higher in the co-culture than in At and Ts monocultures. After seven days of fermentation, cellulose from the mixed substrate was completely degraded by At monoculture and co-culture of At and Ts, while only 45.2% of cellulose was degraded by Ts monoculture. In the meantime, co-culture exhausted all xylan present in the mixed substrate, but At and Ts monocultures degraded only 56.8% and 69.9% of xylan, respectively, suggesting that co-culture of At and Ts had the synergistic effect on substrate degradation caused by synergy of their cellulase systems. However, to facilitate analysis of the utilization of substrates, the mixture of cellulose and xylan instead of lignocellulosic biomass was used to ferment in this study. Thus, if At and Ts were directly grown on unpretreated lignocellulosic biomass such as corn stover, the utilization rate of substrates would decrease dramatically due to the accessibility of cellulase systems, despite having a high synergistic effect on substrate degradation.

Furthermore, the soluble sugar in the fermentation broth was monitored by the DNS method (Figure 3c). It was indicated that the sugar concentration of At monoculture was increased as the duration of fermentation, while the sugar of Ts monoculture and co-culture was kept at an extremely low level after three days of fermentation. It is because At cannot utilize pentose, and continual accumulation of pentose sugars, most of which are xylose, was observed in the fermentation broth of At. The results of polysaccharide degradation and sugar utilization indicate another advantage of Ts, which is simultaneous utilization of pentose and hexose (co-utilization). This happens to be a drawback of At in consolidated-bioprocessing of cellulosic ethanol.

Ethanol, acetate, and lactate were the main end products produced by the tested co-cultures of At and Ts. Variation in the metabolic product distribution was observed depending upon the substrates and culture combinations. The highest ethanol yield (40.6% of the theoretical maximum) was generated by the co-culture of At and Ts with a cellulose/xylan-to-ethanol conversion rate of 20.7% that is higher than 19.3% and 15.2% of At and Ts monocultures (Figure 3d). Wild-type strains of At typically produce ethanol with a yield of about 10–35% of the theoretical maximum [37], which is consistent with our results of At monocultures. However, the ethanol yield reached more than 40% when At was co-cultured with Ts, suggesting that metabolism of xylan by Ts can contribute to ethanol production.

Additionally, formate was not detected in the Ts fermentation broth, suggesting that Ts lacks pyruvate formate lyase (Pfl), which catalyzes pyruvate to formic acid and acetyl coenzyme A. Some studies have demonstrated that a *pfl* mutant produced more ethanol than the parent strain [38]. Thus, Ts can serve as a good partner for production of ethanol in co-fermentation with At.

However, acetate (14.7 mM) and lactate (16.3 mM) were also observed in the co-culture broth, suggesting that cellulose was degraded through branched pathways other than producing ethanol. Therefore, At and Ts need to be engineered or further evolved to improve their ethanologenic capabilities by blocking the branched organic acid pathways. For example, in a metabolically engineered thermophilic bacterium lacking the pathway producing organic acid, the conversion rate of a monosaccharide mixture to ethanol reached 38% and 37.0 g/L ethanol was ultimately produced [39].

## 4. Discussion

Lignocellulosic represents the most abundant biomass with potential for biofuel, and chemical production on earth and has emerged as a promising alternative for reducing our dependence on fossil fuels. Lignocellulolytic microorganisms are widely distributed in the Bacteria, Archaea, and Eukarya, such as Clostridium species, white-rot basidiomycete fungi, and rumen microbial ecosystem. Among them, anerobic, thermophilic, and cellulolytic bacteria have been regarded as CBP candidate organisms because they offer a suitable fermentative temperature (60 °C), lowered energy requirements, minimized contamination risk, and maximized cost effectiveness [11,40]. In addition, some such strains can efficiently degrade lignocellulosic substrates by employing a high molecular weight, multienzyme complexes-cellulosome, containing several glycoside hydrolases [41,42]. Thus, we analyze the potential of At and Ts, two thermophilic and cellulolytic bacteria for lignocellulose utilization in this study.

Lignocellulose is a recalcitrant network of polysaccharides that are highly resistant to enzymatic degradation. Its degradation tends to need interaction of the multiple glycoside hydrolases (GHs), polysaccharide lyases (PLs), and carbohydrate esterases (CEs), in which the collective activity of cellulases is often greater than the sum of the individual enzyme activities. Natural hydrolase systems for lignocellulose degradation have two opposing types: the concentration of cellulosome and dispersion of the free-cellulase system according to their molecular organization [43,44], of which cellulase systems from At and Ts are, respectively, the typical representatives. Many studies showed that the assembled/covalently linked enzymes generally have higher activity for substrate hydrolysis than the corresponding mixture of the disassembled/separated enzymatic units [34,45]. It was also confirmed by the enzyme activity comparation between cellulase systems from At and Ts in a way, in which cellulase systems from At released more sugars than that of Ts under Avicel and corn stover. However, our study revealed that there was a high synergetic effect for degrading, when the cellulase systems from At mixed with that of Ts. It could be well explained by their difference in direction of substrate deconstruction. Zajki-Zechmeister et al. [46] observed that the cellulosome forced the cellulose fiber degradation into the transversal direction, while free enzymes promoted substrate deconstruction in the opposite (lateral) direction by employing real-time atomic force microscopy. Similar results were also noted in studies of the degradation of soft wood kraft pulp [47]. Consequently, the cellulosome system of At can be regarded as a wedge inserted into the crystalline cellulose and degrading it transversally. In contrast, the noncomplexed cellulase system of Ts acts as shears, degrading amorphous cellulose laterally by cutting the glucose chain section by section. Therefore, degradation of Avicel can be accelerated by synergy of the cellulosome and free-cellulase systems. Thus, the cellulases enzyme efficiency increased by tethering together an assortment of cellulases and related accessory enzymes.

**Figure 4:**
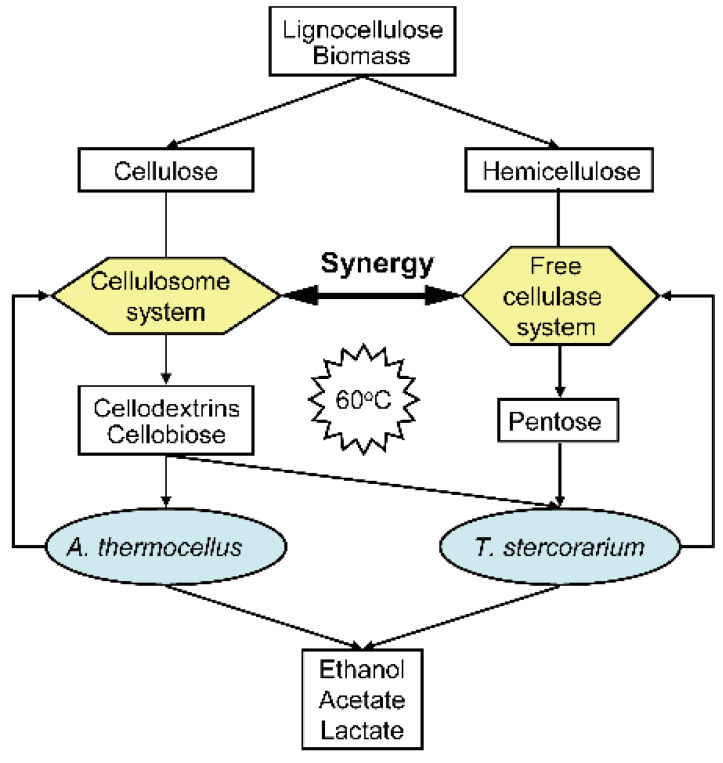
The schematic diagram of CBP employing co-fermentation of *A. thermocellus* and *T. stercorarium*. Lignocellulose biomass can be synergetically hydrolyzed by both cellulase systems of *A. thermocellus* and *T. stercorarium*. The hemicellulose-derived pentoses can be utilized by *T. stercorarium* but not *A. thermocellus*. Cellobiose and cellodextrins are produced from cellulose and can be utilized directly by both *A. thermocellum* and *T. stercorarium*.

CBP requires microorganisms or microbial systems to harbor the capabilities of efficiently degrading lignocellulose and simultaneously yielding chemicals. However, few native microorganisms can directly degrade lignocellulose and efficiently produce ethanol [48,49]. In recent years, the strategy of co-fermentation of cellulolytic and ethanolgenic thermo-anaerobes has attracted much attention due to their symbiotic behavior offering the exchange of metabolites, improved stability, and a reduced lag phase [11,50]. For example, a co-culture of cellulolytic At and ethanolgenic *Clostridium thermolacticum* achieved enhanced ethanol yield, which is 80% of the theoretical maximum during cellulose fermentation [51]. Another study showed that a co-culture of *A. thermocellum* and *Thermoanaerobacter pseudethanolicus* is effective for improved ethanol production during batch fermentation [52,53]. Here, we proposed a CBP strategy by employing a co-culture of At and Ts (Figure 4), in which lignocellulose biomass can be synergetically hydrolyzed by both cellulase systems of At and Ts, and cellobiose and cellodextrins are produced from cellulose and can be utilized directly by both At and Ts while hemicellulose-derived pentoses can be only utilized by Ts. Compared to the co-culture of cellulolytic and ethanolgenic bacteria, it has several advantages. First, judging from the technical route, fermentations of At and Ts are parallel from cellulose to ethanol, during which the mutual independence pathways make their interaction more easily. However, co-fermentation of At and ethanolgenic bacterial is stepwise, during which ethanol fermentation is limited by the rate of cellulose hydrolysis performed by At. Second, co-culture of At and Ts can rapidly hydrolyze lignocellulose due to the synergistic effect between cellulase systems of Ts and At. Third, both At and Ts preferentially utilize cellodextrins and cellobiose by intracellular cellobiose/cellodextrin phosphorylase and generate glucose-1-phosphate, which can save one ATP molecule per reaction [54]. Thus, it is more efficient to take up the oligomers derived from lignocellulose rather than degrade extracellularly and transport the monomer sugars. These findings demonstrate the significance of the cellulase systems in At and Ts, suggesting rational strategies to maximize synergy of cellulase–cellulase systems, cellulase system–microbe and microbe–microbe, which provide deep insights into CBP system developing and engineering. Recently, the whole genome sequencing and annotation and development of genetic manipulation techniques have not only deepened the understanding of the ethanol metabolic networks of thermophilic cellulolytic Clostridia but also facilitated the engineering of cellulolytic Clostridia for ethanol production by CBP [55,56].

## 5. Conclusions

In this study, we compared the cellulase systems from *A. thermocellus* and *T. stercorarium*, which are good at degrading cellulose and hemicellulose, respectively. The two types of cellulase systems were demonstrated to have a synergistic effect, especially against corn stover, with a synergy degree up to 1.87. Ethanol yields of the co-culture of *A. thermocellus* and *T. stercorarium* were nearly doubled those of the monocultures with a cellulose-to-ethanol conversion rate up to 20.7%, which should serve as valuable cases to improve the degradation and fermentation ability of Avicel in actual application.

## Figures and Tables

**Table 1:** Enzyme components of cellulase system from *A. thermocellus* and *T. stercorarium*.

Family	Number		Known Activities
	At ^a^	Ts	
GH1	2	0	exo-β-glucosidase
GH2	1(1)	7	β-galactosidase
GH3	2	5	β-glucosidase/xylosidase
GH4	0	1	α-galactosidase
GH5	10(8)	0	β-mannanase/xylanase/endo-β-1,4-glucanase
GH8	1(1)	0	endo-β-1,4-glucanase
GH9	16(15)	1	Processive endoglucanase/cellobiohydrolase/endo-β-1,4-glucanase
GH10	5(4)	4	β-1,4-xylanase/xylanase
GH11	1(1)	1	endo-1,4-β-xylanase
GH13	2	6	1,4-α-glucan branching enzyme/α-glycosidase
GH15	1	1	
GH16	2(1)	0	β-1,3-1,4-glucanase/lichenase
GH18	4(1)	2	Chitinase
GH23	2	2	
GH26	3(3)	1	β-mannanase/endo-β-1,4-glucanase/β-1,4-xylanase
GH27	0	1	
GH28	0	2	
GH29	0	1	
GH30	2(2)	0	glucuronoxylan xylanohydrolase
GH31	0	1	
GH35	0	1	β-galactosidase
GH36	0	2	α-galactosidase
GH38	0	1	
GH39	1(1)	1	β-xylosidase
GH43	6(6)	8	α-L-arabinofuranosidase/exo-β-1,3-galactanase/β-xylosidase/arabinosidase
GH44	1(1)	0	
GH48	2(1)	1	exo-cellulase
GH51	1	1	α-L-arabinofuranosidase
GH53	1(1)	1	endo-β-1,4-galactanase
GH67	0	1	α-glucuronidase
GH74	1(1)	0	Xyloglucanase
GH78	0	1	α-L-rhamnosidase
GH81	1(1)	0	β-1,3-glucanase
GH88	0	1	
GH94	3	2	Cellobiose/cellodextrin phosphorylase
GH95	0	1	
GH105	0	5	
GH106	0	3	α-L-rhamnosidase
GH112	0	1	
GH115	0	1	
GH124	1(1)	0	endo-β-1,4-glucanase
GH126	1	0	
GH127	0	2	
GH130	1	1	
GH140	0	1	
GH141	1	0	xylanase E
GH154	0	2	
NC	1	1	

^a^ No. of cellulosomal components of *A. thermocellus* was indicated in parentheses according to annotated genome DNA.

**Table 2:** Comparison of enzymatic activities of cellulase systems from *A. thermocellus* and *T. stercorarium*.

Substrate	Activity in IU/mg	
	At	Ts
Avicel PH101	0.050 ± 0.038	0.021 ± 0.043
Carboxymethylcellulose	0.549 ± 0.073	0.472 ± 0.136
Amorphous cellulose	0.543 ± 0.054	0.279 ± 0.048
Waterman No. 1^#^ filter paper	0.047 ± 0.004	0.036 ± 0.011
Xylan from oat spelt	0.414 ± 0.108	0.800 ± 0.098
Pectin from citrus peel	0.162 ± 0.010	0.185 ± 0.021
Cellobiose	0.101 ± 0.007	0.011 ± 0.001

**Table 3:** Product profiles of *A. thermocellus* and *T. stercorarium* against Avicel and corn stover.

Sugar Con. (mM)		Cellulase Systems		
		At	Ts	At + Ts ^a^
Avicel	Glucose	2.13 ± 0.057	0.97 ± 0.010	2.30 ± 0.072
	Cellobiose	2.51 ± 0.070	0.92 ± 0.015	3.28 ± 0.093
Corn stover	Glucose	1.03 ± 0.056	0.80 ± 0.025	1.04 ± 0.032
	Xylose	0.35 ± 0.015	0.91 ± 0.020	0.71 ± 0.071
	Cellobiose	1.92 ± 0.036	0.69 ± 0.026	2.34 ± 0.035

^a^ Mixed the cellulase systems from At and Ts with the ratio of 3:2.

## Data Availability

Not applicable.
